# Megaprosthesis in Knee Arthroplasty Revision for Non Oncologic Patient: A Narrative Review

**DOI:** 10.3390/jcm15208126

**Published:** 2026-10-20

**Authors:** Francesco Schettini, Miriam Grassi, Marco Mattia Larghi, Alfonso Manzotti

**Affiliations:** 1 School of Medicine and Surgery, Residency Program in Orthopaedics and Traumatology, University of Milan, 20122 Milan, Italy; francesco.schettini@unimi.it; 2 Orthopaedics and Traumatology, Luigi Sacco Hospital ASST FBF SACCO, 20157 Milan, Italy; larghi.marco@asst-fbf-sacco.it (M.M.L.); manzotti.alfonso@asst-fbf-sacco.it (A.M.)

**Keywords:** revision knee surgery, megaprostheses, reconstructive surgery, periprosthetic fracture (PFF), periprosthetic joint infection (PJI), massive bone loss

## Abstract

In knee reconstructive surgery, total knee arthroplasty (TKA) is a well-established and successful procedure, with a substantial worldwide increase in the number of procedures performed, driven by an aging population and expanding indications. Consequently, a growing challenge in modern arthroplasty surgery is the progressively increasing cumulative burden of complex revision TKA (rTKA), which often involves severe periprosthetic bone loss, a history of multiple previous surgical failures, periprosthetic joint infections (PJI), periprosthetic fractures (PPFs), or ligamentous instability with disruption of the extensor mechanism. In these highly compromised scenarios, megaprostheses, originally developed for limb salvage following musculoskeletal tumor resections in orthopedic oncology, have emerged as a valuable salvage technique and have been progressively adapted for demanding non-oncological reconstructive revision procedures. Despite the increasing adoption of megaprostheses, clinical decision-making in non-oncological rTKA remains a topic of ongoing debate. A clear consensus regarding precise clinical indications, advantages and disadvantages, and long-term survival rates compared with alternative reconstructive techniques is still evolving. The aim of this review article is to specifically analyze the current literature regarding the indications, outcomes, and rates and types of complications associated with megaprosthetic reconstruction in revision total knee arthroplasty, providing orthopedic surgeons with an evidence-based framework for patient selection and surgical planning.

## 1. Introduction

Total knee arthroplasty (TKA) is one of the most successful and cost-effective procedures in orthopedic reconstructive surgery, significantly improving quality of life and joint function in patients with severe osteoarthritis. However, as the global volume of primary TKAs continues to increase, driven by an aging population and expanding indications, the cumulative burden of complex revision TKA (rTKA) has increased proportionally [1]. Revision procedures present substantial technical challenges, particularly in cases involving severe periprosthetic bone loss, previous failed hardware/implant, multiple prior surgical failures, periprosthetic joint infection (PJI), periprosthetic fractures (PPFs), or ligamentous instability with disruption of the extensor mechanism [2]. However, in the setting of extensive bone loss (classified as Anderson Orthopaedic Research Institute [AORI] Type III defects), conventional revision strategies—such as structural allografts, porous metal cones, and metaphyseal sleeves—may be inadequate to restore structural integrity and achieve stable, durable implant fixation. In these highly compromised scenarios, megaprostheses have emerged as a vital salvage technique. Originally developed for limb salvage following musculoskeletal tumor resections in orthopedic oncology, megaprosthetic systems have been progressively adapted for complex non-oncological reconstructive revision surgeries [3]. Megaprostheses eliminate reliance on deficient metaphyseal and epiphyseal bone stock, relying instead on diaphyseal fixation with constrained hinged articulations. While these implants offer immediate structural stability, early weight-bearing, and predictable functional restoration, they are associated with non-negligible complication rates, including mechanical failures, soft-tissue complications, and a notably elevated risk of deep infection, with an overall infection-free implant survival rate of 42.0% [4]. Despite their growing adoption, clinical decision-making regarding the use of megaprostheses in non-oncological rTKA remains a topic of ongoing debate, and a clear consensus regarding precise clinical indications, long-term survival rates, and risk-benefit profiles compared with alternative reconstructive techniques is still evolving.

For this purpose, an ideal candidate for megaprosthetic implantation requires a thorough preoperative assessment, including host classification associated with a risk stratification process discussed with the patient. Likewise, a comprehensive imaging protocol should be implemented, including at least a full-length weight-bearing radiograph, a weight-bearing knee radiograph with an axial patellar view, and a CT scan of the knee, to accurately define the surgical plan.

The aim of this review article is to comprehensively synthesize the current literature regarding the specific indications, advantages, and disadvantages of megaprosthetic reconstruction in revision total knee arthroplasty in non-oncological patients, providing orthopedic surgeons with an evidence-based framework for patient selection and surgical planning.

## 2. Methods and Scope of the Review

This narrative review summarizes the current state of the art research regarding the specific indications, surgical principles, major modes of failure, and antimicrobial strategies associated with megaprosthetic reconstruction in peri-knee revision surgery, providing an evidence-based framework for patient selection and surgical planning. To ensure methodological transparency, a comprehensive literature search was conducted in PubMed to identify peer-reviewed studies published in English up to April 2026. The search strategy used combinations of keywords, including “knee megaprosthesis,” “distal femoral replacement,” “proximal tibial replacement,” “non-oncological revision TKA,” “periprosthetic joint infection,” and “periprosthetic fracture.” Only studies reporting clinical outcomes, survival rates, and complication profiles in non-oncological knee reconstruction using megaprostheses were selected for this review, while oncological series were excluded or considered solely as a technical reference. Both retrospective cohorts and systematic reviews were analyzed, with emphasis placed on tracing findings back to the original primary research studies to minimize secondary citation bias. During the preparation of this manuscript, the authors used the sixth version of ChatGPT (OpenAI, San Francisco, CA, USA) solely for language refinement.

### 2.1. Indications

The broad spectrum of non-oncological indications is well documented in the literature [5,6,7]. Vaishya et al. systematically reviewed non-neoplastic distal femoral replacements (DFRs) across 54 cases (mean age 75 years, mean follow-up 43 months), observing that substantial bone loss resulting from failed primary arthroplasties was the primary indication for megaprosthesis implantation (55.5%), with PJI identified as the leading complication (18.5%) [6,7]. Similarly, Korim et al. analyzed 241 non-neoplastic DFR cases across nine studies (mean follow-up 3.3 years) and reported a 17% reoperation rate, primarily due to infection, followed by aseptic loosening and mechanical failure [8] (Table 1).

### 2.2. Periprosthetic Fracture

**Figure 1:**
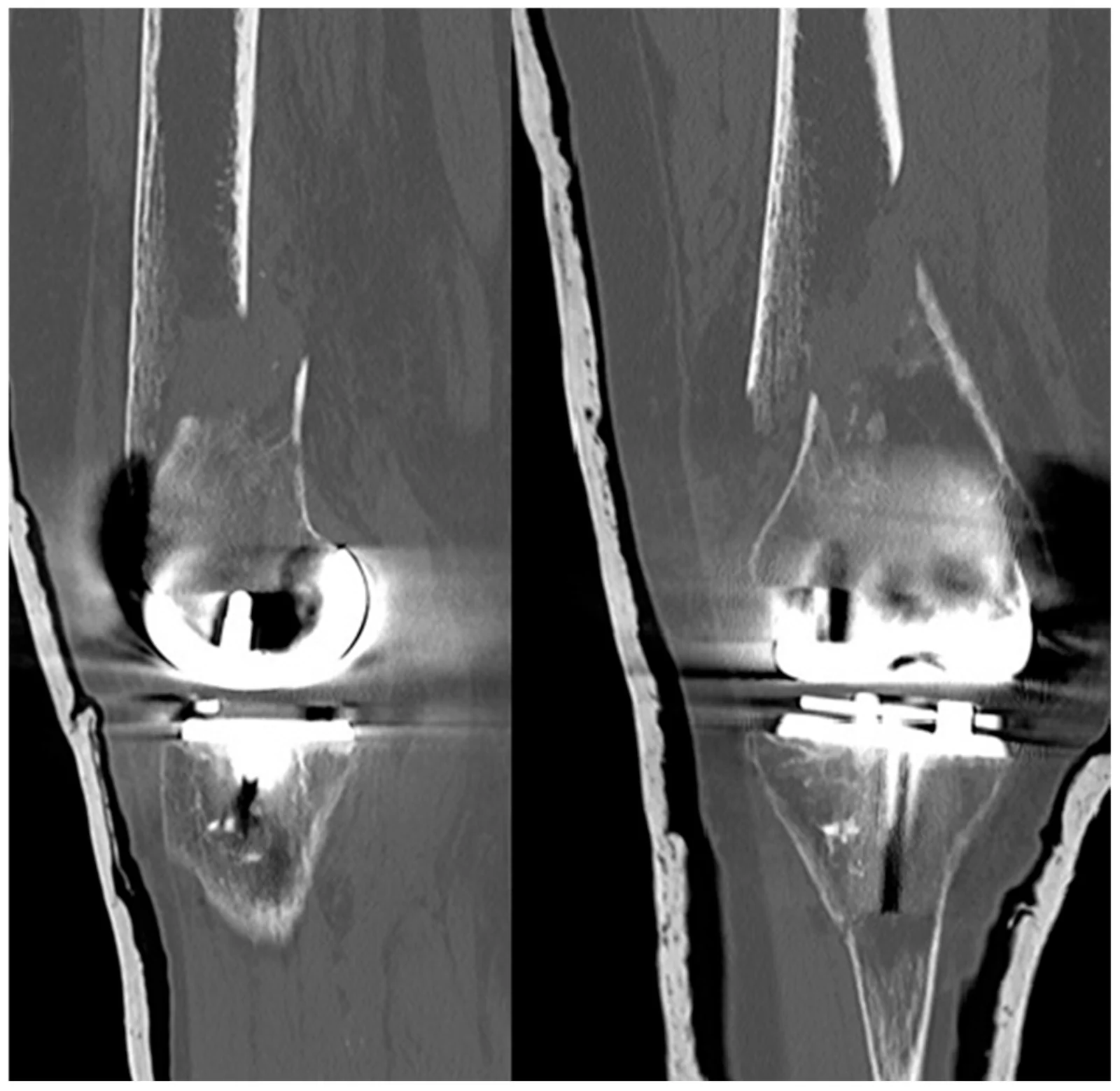
Evidence of periprosthesis fracture associated with bone loss in an 84 year-old lady V.3 (distale femur) -B.3 (implant instability with high bone loss) according to Unified Classification System for Periprosthetic fractures UCPF).

**Figure 2:**
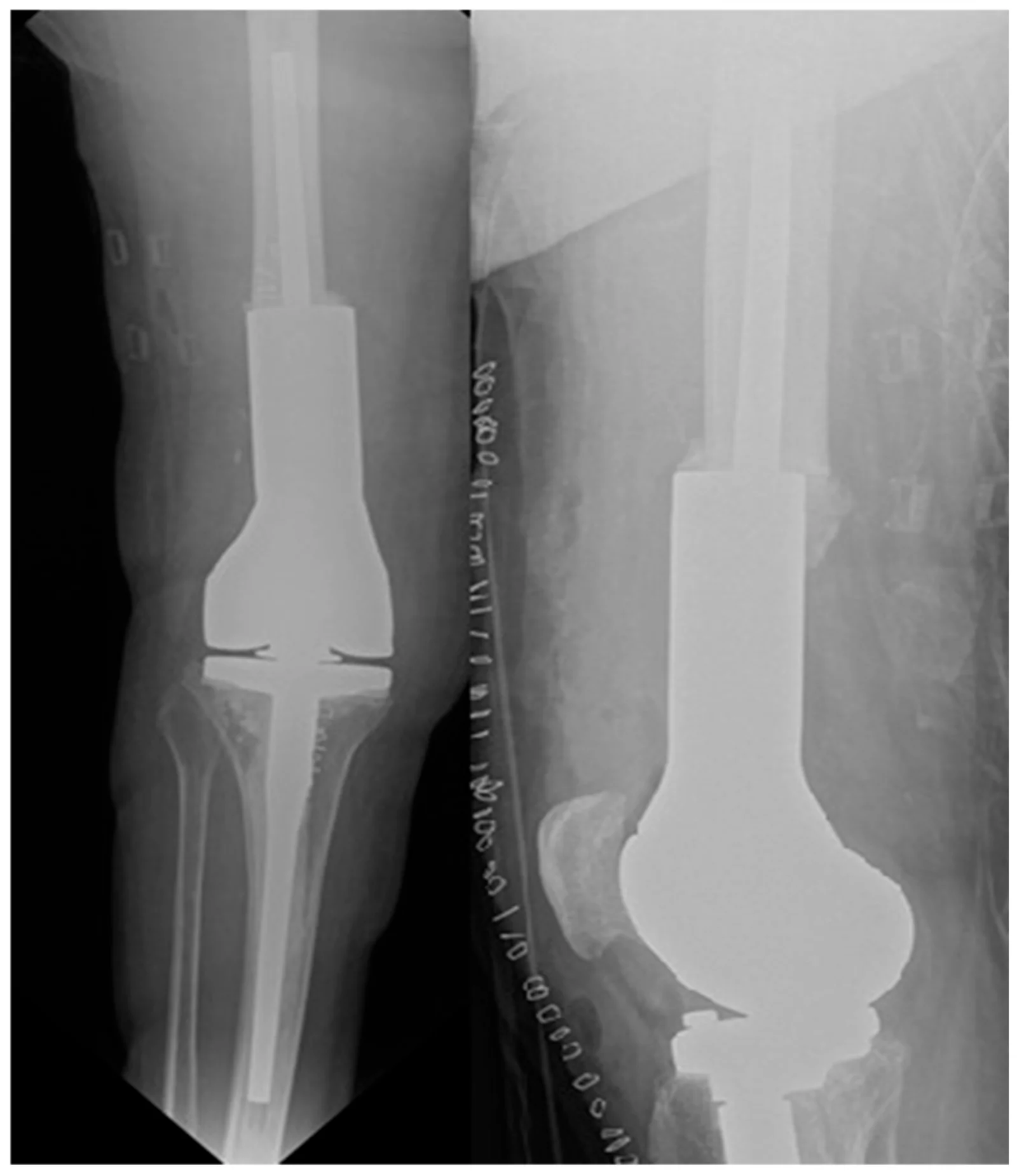
Postoperative X-rays in megaprosthesis in periprosthetic fracture in the same patient of Figure 1, treated at Ospedale Luigi Sacco ASST Fatebenefratelli—Sacco by the senior surgeon.

Among the non-oncological indications, periprosthetic fractures (Table 2) (Figure 1) in elderly patients with severe comminution or massive osteolysis that precludes osteosynthesis represent a “practical” indication for megaprosthetic reconstruction, which may constitute the only viable reconstructive option to restore function and enable a rapid return to normal daily activities [4,5,6,7,8,9,10,11,12,13] (Figure 2). Likewise, in the context of traumatic non-unions, Henderson et al. evaluated the complications of locked plating for distal femoral fractures and reported a 20% non-union rate, together with a 27% reoperation rate in patients over 60 years old, suggesting this age as a potential threshold beyond which megaprosthetic replacement may become advantageous [11]. Furthermore, Windhager et al. reported clear benefits of megaprosthetic reconstruction for both distal femoral (Subtype 3) and proximal tibial (Felix Type 1B) periprosthetic fractures, particularly when accompanied by implant loosening [14].

### 2.3. Massive Bone Loss/Aseptic Revision

Beyond fracture management, megaprostheses are also indicated in complex revision cases involving catastrophic bone loss that cannot be adequately addressed using allografts, augments, or cones. Such severe defects typically result from:

(a) Polyethylene wear and extensive osteolysis;
(b) Cumulative bone loss across multiple revision procedures;
(c) Traumatic or complicated implant extraction;
(d) Deliberate bone resection during the management of periprosthetic joint infections (PJI);
(e) Failed hardware/implant causing bone loss [14,15,16,19,20,21,22,23].

### 2.4. Periprosthetic Joint Infection

**Figure 3:**
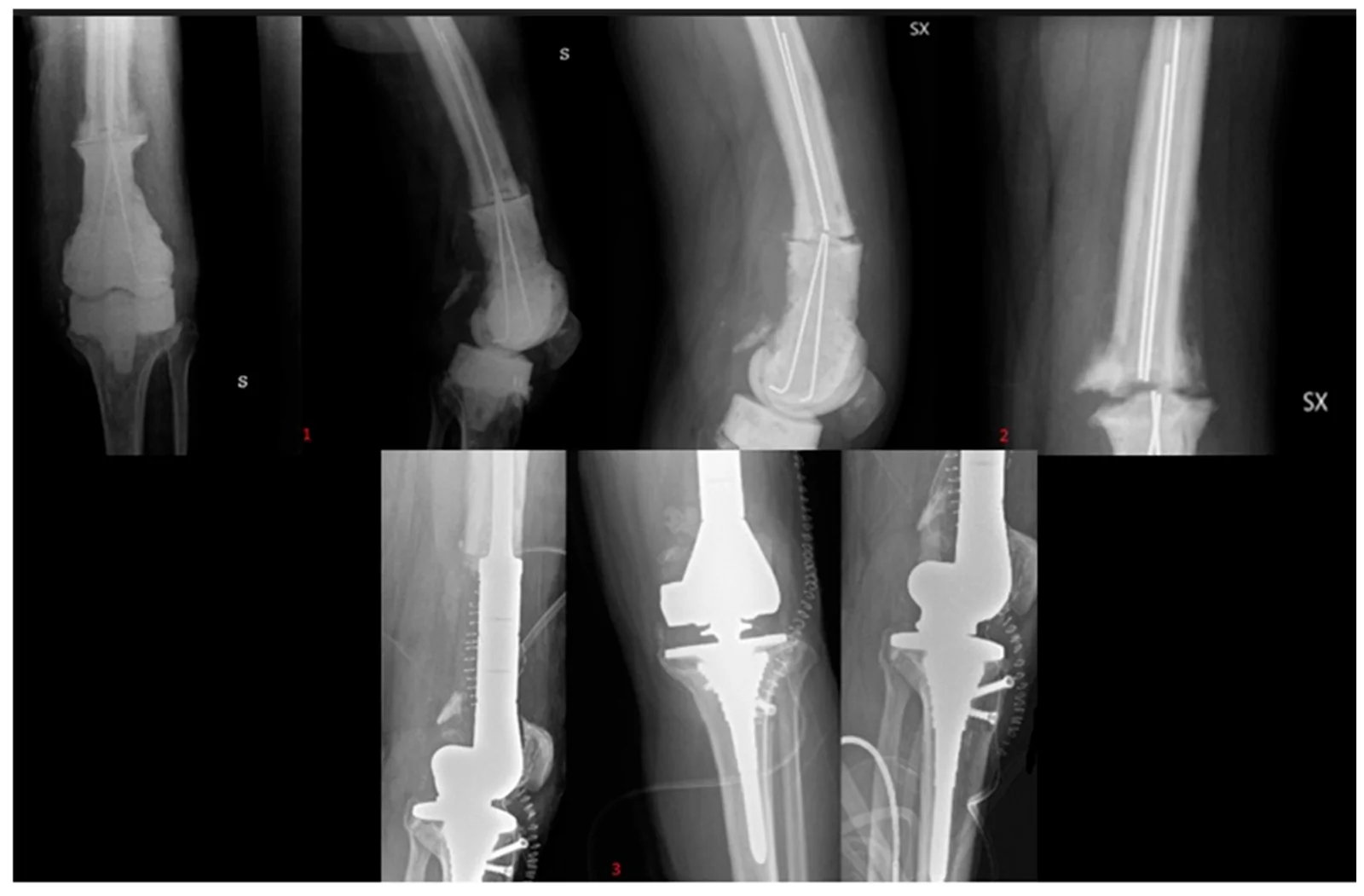
Revision with megaprosthesis (number 3) in periprosthetic joint infection (PJI) in a complicated case sequent rupture antibiotic-loaded cemented knee spacer (number 1 and 2), treated at Ospedale Luigi Sacco ASST Fatebenefratelli—Sacco by the same senior surgeon.

Megaprostheses represent a critical limb-salvage option in end-stage infection scenarios (Table 3)—such as septic non-union or periprosthetic joint infection (PJI) (Figure 3)—allowing surgeons to avoid amputation. Although the large metallic surface area of these implants increases the potential risk of infection recurrence, a staged procedure allows for more extensive bone resection and debridement compared with conventional reconstructive procedures, effectively eradicating foci of osteomyelitis with reported success rates of approximately 82.8% [14]. Press-fit uncemented fixation is generally preferred in younger patients, whereas cemented fixation offers a distinct advantage in older patients, particularly when antibiotic-impregnated cement is used, permitting immediate full weight-bearing—even though the optimal stem fixation method remains a subject of ongoing debate [2,3].

### 2.5. Economical Assessement

From a purely economic perspective, it should be considered that, in cases of PJI, the cost of revision surgery following primary TKA may range from 13,910 USD to 38,109 USD (in the case of septic revision performed using a two-stage approach), whereas in cases of septic re-revision, costs range from 66,629 to 81,938 USD. The main contributors to these costs are preoperative expenses (33%), the cost of the revision prosthesis (28%), and the cost of hospital stay (22%) [24,25]. Likewise, when considering TKAs associated with periprosthetic fractures, the costs of osteosynthesis procedures using dedicated plates and related instrumentation have been reported to be approximately 12,000 USD, with longer hospital stays and a rehabilitation pathway requiring greater patient assistance [17], resulting in a delayed return to an almost normal social life. This brief cost analysis highlights that, even when considering the economic aspect alone, there is no clear preference for megaprostheses over osteosynthesis techniques in cases of periprosthetic fractures, nor for megaprostheses over further revision procedures using revision prostheses in cases of septic or aseptic loosening. Therefore, the main factors guiding the choice among the different therapeutic alternatives appear to be the clinical scenario and the surgeon’s experience.

### 2.6. Surgical Principles

**Figure 4:**
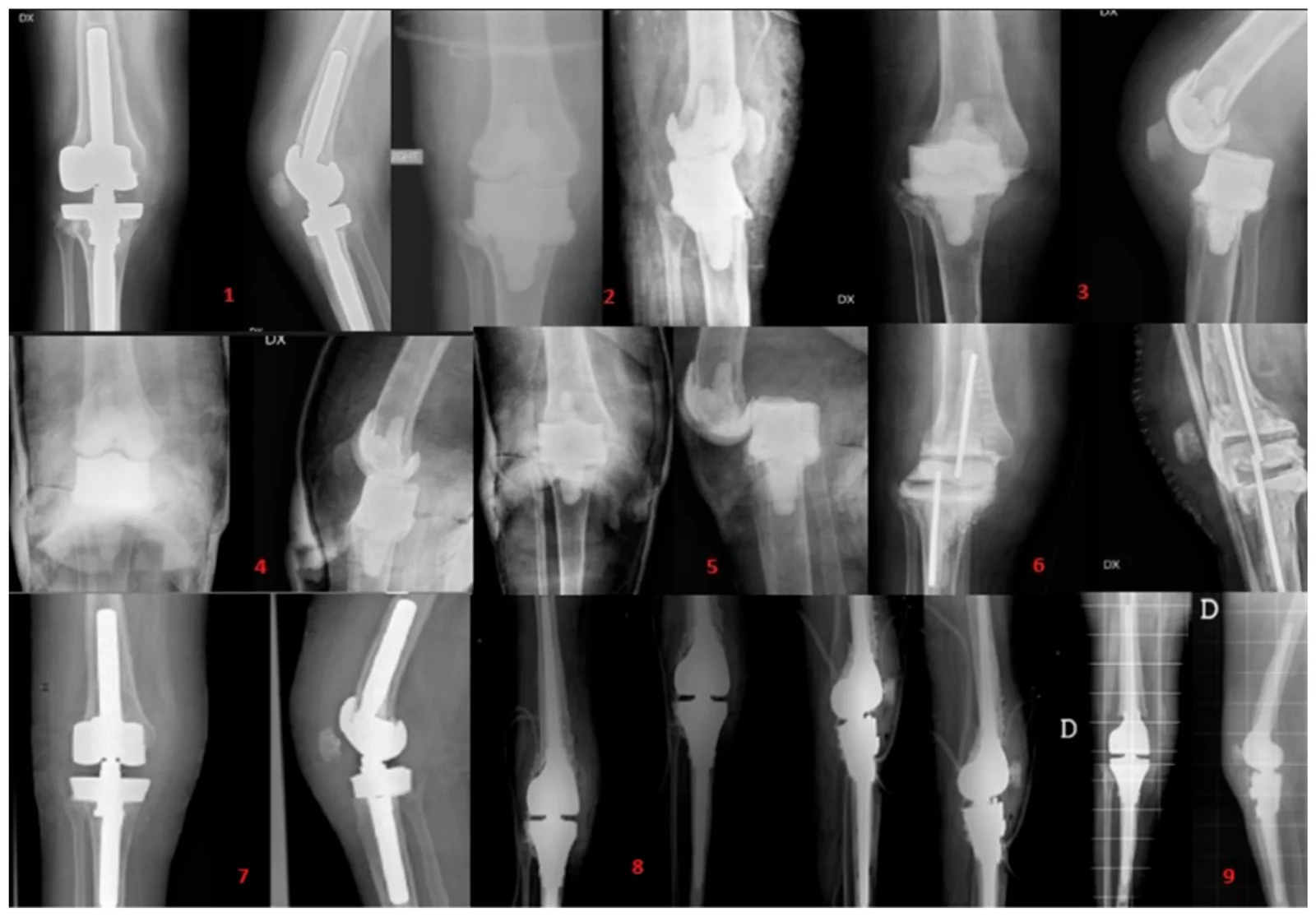
Megaprosthesis in periprosthetic joint infection (PJI) with follow-up at 8 years (number 9) during the postoperative course 2 episodes of dislocation (number 3, 4, and 5). of the antibiotic-loaded cemented knee spacer with tissue loss and surgical wound distress, recurrence of infection, leading to a one-stage revision with megaprosthesis (number 7). New revision (number 8 and 9) in the same patient. All surgeries has been made at Ospedale Luigi Sacco ASST Fatebenefratelli—Sacco by the same senior surgeon.

Several authors have reported their experience with megaprostheses around the knee in non-neoplastic conditions, highlighting their potential advantages in highly selected cases despite high complication rates. However, the precise balance between risks and benefits remains poorly defined, and the available literature is limited to small retrospective series analyzing complex total knee arthroplasty (TKA) revisions [5,6,7]. Several authors have emphasized the clinical benefits of early mobilization in elderly patients with non-neoplastic peri-knee conditions, noting that a definitive, “one-shot” surgical procedure often represents the most advantageous solution for frail individuals [17,18,25]. This is echoed by Holl et al. in one of the largest series of TKA revisions with megaprostheses; the authors highlighted distinct advantages for elderly patients with limited life expectancy—noting an unrelated 45% overall mortality rate—and advocated limb salvage whenever feasible to minimize psychological distress [26]. Nevertheless, they stressed that these highly complex procedures should be performed exclusively in specialized tertiary centers experienced in both complex revision arthroplasty and megaprosthetic techniques (Figure 4). Although megaprosthetic reconstruction is widely considered a radical solution, Calori et al. [27] argue that this approach allows surgeons to achieve optimal correction of lower limb deformities, effectively aligning clinical outcomes with patient expectations while maximizing functional recovery. Supporting this rationale, a recent paper by De Marco et al. [28] concluded that megaprosthetic knee implants represent a valuable therapeutic option for periprosthetic knee fractures. Furthermore, by evaluating and managing both trauma patients and post-traumatic patients with severe deformities and/or significant bone loss according to the same rigorous principles applied to oncological patients, this approach significantly reduces postoperative immobilization and length of hospital stay [27]. Beyond the constrained stability and large implant dimensions that allow surgeons to address unreconstructable bone loss and severe instability, recent technical advancements have further expanded the indications for megaprostheses, with the aim of reducing complication rates. Improved component modularity helps address extensor mechanism dysfunction, offering intraoperative flexibility through reinforced patellar tendon reattachment using dedicated tibial plates or, in extreme cases, direct conversion to knee arthrodesis using modular pins [5,14,19]. Finally, the availability of both uncemented and long cemented stems, together with custom-made porous metaphyseal cones designed to treat tibial and femoral bone defects, represents a promising development for facilitating early weight-bearing with cementless implants while ensuring stable fixation in both healthy and osteopenic bone [17,29].

To summarize the findings reported in the literature, the main advantages of megaprostheses in TKA revisions performed in specialized centers include:

- Management of massive bone loss and severe soft-tissue instability;
- Treatment of complex periprosthetic fractures (Su Type 3 distal femoral and Felix Type 1B proximal tibial fractures), even in the presence of loose implants, in elderly patients (aged at least 67 years);
- Effective fixation in severely osteoporotic bone in elderly patients (aged at least 73 years);
- Flexible fixation options (cemented vs. uncemented stems) to achieve immediate stability and early mobilization;
- Management of extensor mechanism dysfunction through modular tibial attachments or conversion to modular arthrodesis;
- Prevention and control of infection in septic revisions using advanced antibacterial coatings;
- Reduced psychological distress and surgical burden in frail elderly patients through a definitive “one-shot” limb-salvage procedure.

### 2.7. Complications and Salvage Strategies

Despite these immediate benefits, the clinical utility of megaprostheses is severely compromised by historically high complication rates and catastrophic modes of failure. A comprehensive 2023 literature review encompassing 56 clinical studies in non-oncological settings confirmed that approximately 20% of all megaprosthetic implants experience major complications [30]. Because of these biological and mechanical risks, several authors have advocated restricting these highly complex procedures exclusively to specialized tertiary centers experienced in both complex revision arthroplasty and megaprosthetic techniques [11,31]. More recently, to standardize outcome analysis, Henderson et al. categorized megaprosthetic complications into five distinct failure modes (Table 4):

- **Type 1 (Soft-Tissue Failure):** Functional failure may arise from the extensive dissection or reattachment of major muscle insertions (e.g., gastrocnemius, iliotibial tract, patellar tendon). Henderson et al. observed that soft-tissue complications accounted for 12% of all failures, with an absolute incidence of 3% [11]. Furthermore, volumetric conflict increases the anterior femoral offset, generating unphysiological shear stresses and high-tension vectors during knee flexion, potentially causing severe patellar maltracking, patellofemoral impingement, or chronic tendon rupture [1,3]. To mitigate these risks, surgeons must employ complex reconstructive strategies, using synthetic meshes (e.g., Marlex) or complete extensor mechanism allografts to distribute tensile forces and promote host-tissue ingrowth;
- **Type 2 (Aseptic Loosening):** Affecting 5–10% of megaprostheses overall, aseptic loosening disproportionately impacts DFRs. To mitigate high interface forces, anatomically designed uncemented femoral stems providing robust primary fixation are recommended in combination with rotating-hinge mechanisms;
- **Type 3 (Implant Fracture):** Structural breakage occurred in 6.3% of DFRs in early series. However, modern implant designs have significantly reduced this incidence. For example, an evaluation of three successive generations of Stryker megaprosthetic systems (KMFTR, HMRS, and GMRS) demonstrated that the stem breakage rate decreased from 10.5% in the first generation to 3.5% in the second, reaching 0% with the third generation;
- **Type 4 (Infection):** Depending on the cohort and clinical scenario, PJI remains a major challenge, with reported rates ranging from 5% to 40%. The incidence progressively increases when a megaprosthesis is used as a salvage procedure following a previous septic failure in an already severely compromised biological environment [3], as also emphasized by Berger et al. [31].

### 2.8. Antimicrobial Prevention Strategies

A promising strategy for reducing the infection rate associated with megaprosthetic implants is the use of antibacterial coatings. Several potential coating solutions for combating PJIs in megaprostheses have been reported in the literature, with encouraging results in initial trials, although further research and technological development are required [30,32].

Silver coatings reduce the incidence of PJI through the antibacterial activity of silver. Three different silver coatings are available: MUTARS^®^ (Implantcast GmbH, Buxtehude, Germany), Agluna^®^ (Accentus Medical, Didcot, UK), and PorAg^®^ (Waldemar Link GmbH & Co., KG, Hamburg, Germany). A 2021 meta-analysis analyzed infection rates and silver-related complications, finding that the infection rate after revision surgery was 13.7% in patients with silver-coated implants and 29.2% in those with uncoated megaprostheses, demonstrating a statistically significant benefit of silver coatings in preventing infections [33]. In general, the use of MUTARS^®^ has resulted in an almost consistent reduction in the incidence of primary PJI, although limited data are available regarding its effectiveness in revision procedures. The results obtained with Agluna^®^ in both primary and revision implants have been inconsistent. Conversely, PorAg^®^ has demonstrated effectiveness in both PJI prevention and, particularly, revision procedures performed for PJI. Local argyria was reported in 2.2% of patients, whereas no systemic complications were described, including no relevant increases in blood silver concentrations, which were consistently reported to remain well below the toxicity threshold [33].

Another surface treatment that has been clinically investigated in megaprostheses is an antibiotic-loaded hydrogel coating (Defensive Antibacterial Coating–DAC^®^). Despite the limited number of studies available in the literature, one study has demonstrated a reduction in early surgical site infections in a series of tumor megaprostheses [34]. Furthermore, a single case report described the use of DAC^®^ hydrogel in combination with a bacteriophage to treat a megaprosthesis infection, demonstrating the feasibility of using bacteriophages incorporated into a hydrogel to treat knee megaprosthesis infection during a debridement procedure [35].

Megaprostheses can be coated with other biodegradable materials, such as poly(lactic-co-glycolic acid) (PLGA) and poly(D,L-lactide) (PDLLA) polymers, incorporating antibiotics. For example, a degradable gentamicin-loaded PLGA coating for hydroxyapatite (HA)-coated cementless prostheses was developed and shown to significantly reduce infection rates compared with HA-coated implants without gentamicin. Bone cements are also commonly impregnated with various antibiotics, most frequently gentamicin, tobramycin, or a combination of gentamicin and other antibiotics, such as vancomycin. More recently, biologically active coatings incorporating bacteriophages have emerged as a potential application in megaprosthetic reconstruction, highlighting their high specificity, ability to target antibiotic-resistant bacteria, and capacity to disrupt biofilms, as well as providing guidance for their clinical administration. However, clinical adoption remains limited by regulatory barriers, the lack of standardized protocols, and insufficient clinical trial data [36].

The local application of intra-articular vancomycin powder or suspension at the conclusion of arthrotomy has proven to be an effective complementary strategy for reducing the risk of acute periprosthetic joint infection (PJI), ensuring high local antibiotic concentrations without significantly increasing wound-related complications or systemic toxicity [37]. This approach is particularly relevant in complex joint reconstruction and revision surgery, where the prophylactic use of intra-articular antibiotics reduces the overall incidence of deep infections requiring reoperation [38]. Furthermore, experimental studies confirm that targeted doses of intra-articular vancomycin maintain a favorable safety profile for periprosthetic tissues, reducing inflammation and the risk of bacteria-induced osteolysis [39].

## 3. Conclusions

In non-oncological knee surgery, megaprostheses represent an essential limb-salvage option for cases involving massive bone loss, complex periprosthetic fractures, and periprosthetic joint infections (PJIs) in which conventional reconstructive techniques are not viable [17,18,25,26,27,28,29]. Although these procedures provide immediate mechanical stability and enable early full weight-bearing in frail, elderly patients, they remain associated with substantial complication rates, including a non-negligible risk of catastrophic implant failure, recurrent infection, and intractable bone loss, which may ultimately necessitate lower-limb amputation [4,7]. Recent technological advances, including silver-coated surfaces, enhanced component modularity, and flexible stem fixation options, have improved the potential for soft-tissue reconstruction and infection control [37]. Nevertheless, achieving optimal outcomes requires careful patient selection and clearly defined clinical thresholds to balance risks and benefits. Ultimately, given their inherent complexity, non-oncological megaprosthetic reconstructions should be performed exclusively in specialized tertiary centers with extensive expertise in complex revision arthroplasty.

## Figures and Tables

**Table 1:** Primary indications for total knee revision with megaprosthesis and patient’s characteristics.

Primary Indication	Specific Clinical Scenario/Failure Mechanism	Key Clinical Drivers and Patient Characteristics
Failed Total Knee Arthroplasty (TKA)	Substantial bone loss from failed primary/revision TKA (osteolysis, wear, infection, or prior revision steps)	Primary driver for distal femur replacement; severe deficits cannot be reconstructed with standard augments/cones.
Periprosthetic Fracture (Post-TKA)	Severely comminuted fracture or fracture through large osteolytic lesion following TKA	Particularly in geriatric setting where partial weightbearing is infeasible; megaprosthesis is often the sole option.
Multi-Revision Bone Loss	Cumulative step-by-step bone loss across multiple revision procedures or traumatic implant removal	Progressive loss of metaphyseal and diaphyseal bone stock precludes conventional revision components.
Periprosthetic Joint Infection (PJI)	Intended extensive bone resection required for radical infection eradication	Creates massive skeletal defects that require megaprosthetic reconstruction for limb salvage.

**Table 2:** Studies reporting on knee megaprostheses used to treat periprosthetic fractures. Partly studies included only cases with primary megaprosthesis procedures, partly also cases were included that were secondarily converted from osteosynthesis to a megaprosthesis due to non-union.

Author	Year	Femur/Tibia	n	Age	FU	Findings
Keenan [13]	2000	Femur	7	77.5	1 y	6 patients had good/excellent Bristol Knee Score, 1 y Implant survival 86%
Vaishya [6]	2011	Femur	8	74	4 y	KSS 84/88 at latest FU, good ROM
Chen [15]	2013	Femur	49 (36 primary Mega vs. 13 ORIF-to-MEGA)	77 vs. 75	3–4 y	No difference in 2 y mortality: 34 vs. 38%, significantly higher complication rate in ORIF-to-MEGA
Jassim [12]	2014	Femur	11	81	3 y	Mortality 9%, OKS 22.5
Saidi [5]	2014	Femur	7 Mega vs. 9 constraint-Prosthesis vs. 7 Allograft-Implant-Composites	76 vs. 74 vs. 79	0.6 y	No differences in HSS, Mega: shortest hospital stay
Cannon [16]	2015	Femur	27	76.7	3.7 y	29% mortality, 100% implant survival in the remaining
Gan [10]	2018	Femur	7 Mega vs. 8 ORIF	77 vs. 68	3.5 y	No differences in pain and ambulatory function
Darrith [9]	2020	Femur	22 Mega vs. 50 ORIF	76 vs. 72	5.6 y	Mega: worse KSS function, ORIF: more reoperations, no differences in mortality
Risitano [17]	2023	Femur	9	82	2–3 y	Two delayed wound closures and one chronic prosthetic joint infection (PJI) were observed.
Zanchini [18]	2023	Femur	39	69	5	20% complication rate

**Table 3:** Studies reporting on knee megaprostheses used to treat PJI.

Author	Year	N° Patients	Age	FU	Findings
Fiore [3]	2023	45 silver coated	62 y	43 m	Infection-free rate 83%
Vicente [20]	2024	12 uncoated 9 silver coated	75 y (uncoated) 67 y (coated)	48 m uncoated 46 m coated	Infection eradication: 80% uncoated, 82% silver coated. Post operative complications: 33% silver coated, 34% uncoated.
Corona [21]	2018	15	75 y	18m	Infection-free rate 82%
Bartak [22]	2025	36	68 y	6.6 y	Infection-free rate 77%
Smith [23]	2020	14	52 y	2 y	Infection-free rate 58%

**Table 4:** Complications in megaprosthesis according to Henderson classification.

	Megaprosthesis Complications
TYPE 1	Soft-tissue failure (incidence 3%)
TYPE 2	Aseptic loosening (incidence 5–10%)
TYPE 3	Implant fracture (incidence 3–10.5%)
TYPE 4	Infection (incidence 5–40%)

## Data Availability

The data associated with the paper are available from the corresponding author upon reasonable request.
